# Transanal total mesorectal excision (TaTME) versus laparoscopic TME for MRI-defined low rectal cancer: a propensity score-matched analysis of oncological outcomes

**DOI:** 10.1007/s00464-018-6530-4

**Published:** 2018-10-22

**Authors:** Sapho Xenia Roodbeen, Marta Penna, Hugh Mackenzie, Miranda Kusters, Andrew Slater, Oliver M. Jones, Ian Lindsey, Richard J. Guy, Chris Cunningham, Roel Hompes

**Affiliations:** 1 Department Surgery, Amsterdam University Medical Centers, University of Amsterdam, Meibergdreef 9, 1105 AZ Amsterdam, The Netherlands; 2 Department of Colorectal Surgery, Churchill Hospital, Oxford University Hospital NHS Foundation Trust, Oxford, UK; 3 Department of Radiology, Churchill Hospital, Oxford University Hospitals NHS Foundation Trust, Oxford, UK; 4 Department of Surgery and Cancer, Imperial College London, London, UK; 5 Department of Surgery, Catharina Hospital, Eindhoven, The Netherlands

**Keywords:** Rectal cancer, Transanal TME, Conversion, Laparoscopic TME, CRM, Minimal Invasive Surgery, MRI

## Abstract

**Background:**

While a shift to minimally invasive techniques in rectal cancer surgery has occurred, non-inferiority of laparoscopy in terms of oncological outcomes has not been definitely demonstrated. Transanal total mesorectal excision (TaTME) has been pioneered to potentially overcome difficulties experienced when operating with a pure abdominal approach deep down in the pelvis. This study aimed to compare short-term oncological results of TaTME versus laparoscopic TME (lapTME), based on a strict anatomical definition for low rectal cancer on MRI.

**Methods:**

From June 2013, all consecutive TaTME cases were included and compared to lapTME in a single institution. Propensity score-matching was performed for nine relevant factors. Primary outcome was resection margin involvement (R1), secondary outcomes included intra- and post-operative outcomes.

**Results:**

After matching, forty-one patients were included in each group; no significant differences were observed in patient and tumor characteristics. The resection margin was involved in 5 cases (12.2%) in the laparoscopic group, versus 2 (4.9%) TaTME cases (*P* = 0.432). The TME specimen quality was complete in 84.0% of the laparoscopic cases and in 92.7% of the TaTME cases (*P* = 0.266). Median distance to the circumferential resection margin (CRM) was 5 mm in lapTME and 10 mm in TaTME (*P* = 0.065). Significantly more conversions took place in the laparoscopic group, 9 (22.0%) compared to none in the TaTME group (*P* < 0.001). Other clinical outcomes did not show any significant differences between the two groups.

**Conclusion:**

This is the first study to compare results of TaTME with lapTME in a highly selected patient group with MRI-defined low rectal tumors. A significant decrease in R1 rate could not be demonstrated, although conversion rate was significantly lower in this TaTME cohort.

Colorectal cancer remains the third most common malignancy worldwide [1]. The outcomes for patients with rectal cancer have improved since the widespread adoption of total mesorectal excision (TME) [2] and use of neo-adjuvant therapy. Achieving a good quality TME with negative margins is of paramount importance for an optimal oncological resection, reducing the risk of loco-regional recurrence and improving cancer-free survival [3, 4]. Low rectal cancer, however, remains technically challenging. As the distance between the rectal wall and mesorectal fascia tapers towards the anus, the range for error reduces and thus it becomes more difficult to obtain clear margins. Other factors, such as a narrow, irradiated pelvis and obesity, also predict intra-operative difficulties [5]. Consequently, oncological outcomes of low rectal cancer remain inferior compared to more proximal tumors, with higher rates of resection margin involvement and local recurrences [6].

The impact of minimally invasive techniques is evident in regard to short-term peri-operative outcomes, but the effectiveness in terms of oncological results is still a matter of current debate [7–9]. In laparoscopic surgery, the visualization is often limited, particularly in the lower pelvis, due to fixed trocar positions with insufficient angulation in the rigid bony pelvis. This may lead to imprecise distal margin determination with a challenging distal transection, high conversion rates, up to 34% [10], and associated increased risk of loco-regional recurrence [11, 12].

Transanal TME (TaTME), also known as ‘bottom up’ TME, has been pioneered to overcome these difficulties. The approach from below offers clear, direct visualization of the dissection plane, even in a narrow pelvis, allowing a more precise and trauma-free dissection, which should improve the quality of the TME specimen and decrease positive resection margins.

A meta-analysis by Xu et al. showed that short-term outcomes from small cohort studies assessing the benefit of TaTME are promising [13]. However, most comparative studies include rectal tumors at all heights, and therefore potentially underestimate the real benefits of TaTME, as this approach is probably most valuable in lower tumors.

This is the first study to specifically compare TaTME with lapTME for MRI-defined low rectal cancers, according to the LOREC definition for low rectal cancer [14]. By using this definition, based on the anatomical landmark where the tapering of the mesorectum starts, this study focused on a patient group known to pose greater technical difficulty with increased risk of R1 resection (Fig. 1).

**Fig. 1:**
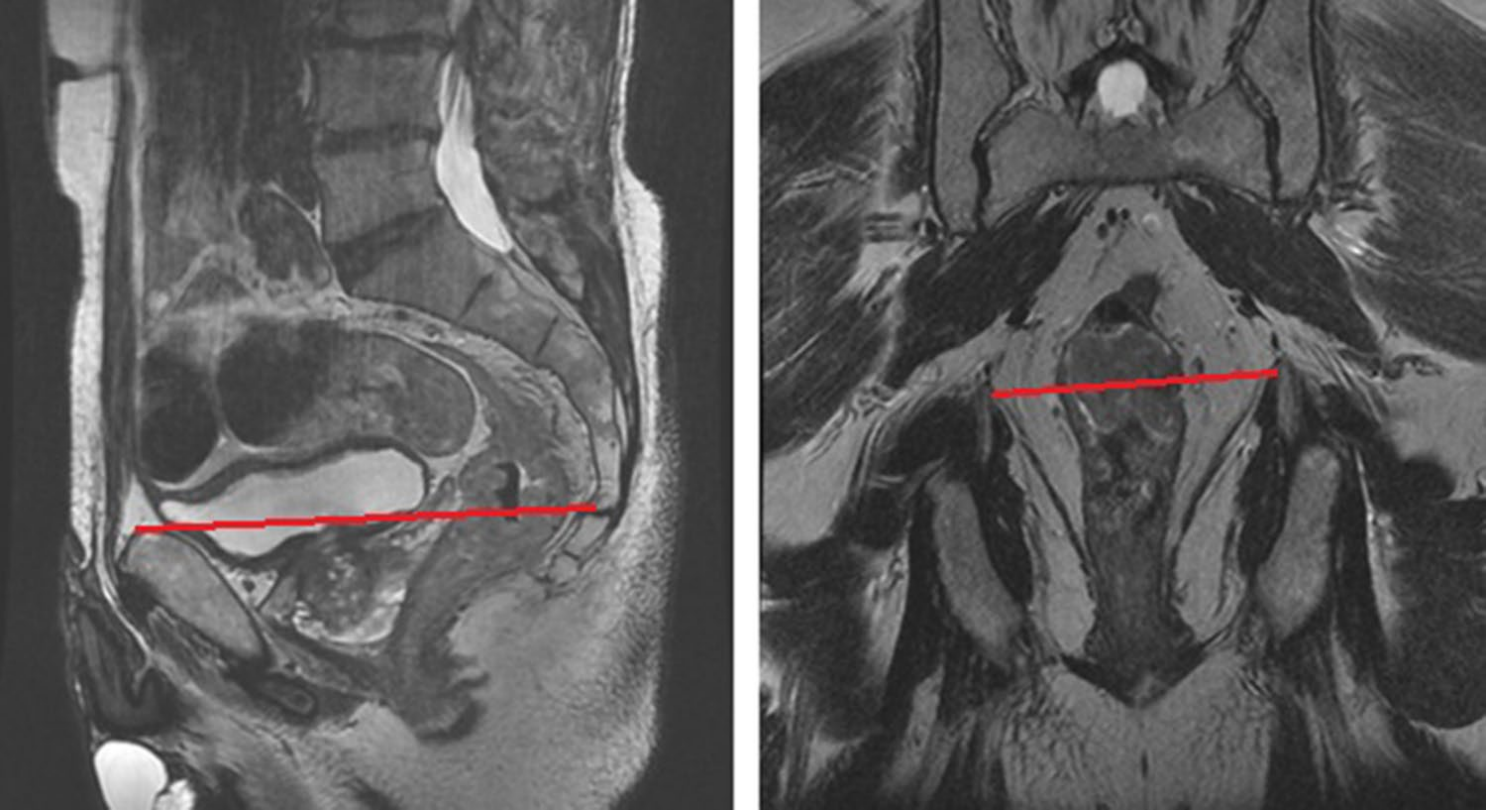
MRI definition of a low rectal tumor (sagittal (left) and coronal (right) T2 MRI-scans showing the line between the pubic bone and the origin of the levator muscles: a tumor below this line was defined as a low rectal cancer)

Our institution adopted and performed TaTME since 2013 as a new surgical approach to treat low rectal cancer. In this propensity score-matching analysis, we aimed to evaluate histopathological and peri-operative outcomes of TaTME versus conventional lapTME, specifically for MRI-defined low rectal cancers.

## Methods

### Study design and patient selection

A single-center prospective cohort study was conducted, comparing TaTME with lapTME. From the first case in June 2013 until July 2017, all rectal cancer patients undergoing TaTME in Oxford were analyzed. All TaTME cases performed in the unit were recorded on the international TaTME registry [15], a prospectively kept database, following the national NICE guidelines. Data were extracted from this database. The control cohort consisted of all rectal cancer cases operated on between August 2006 to July 2017; their data were collected on a prospective institutional database, and cases were further reviewed retrospectively collecting any additional information required for the study.

All patients had biopsy proven rectal adenocarcinoma and underwent curative TME surgery. Benign cases, squamous cancers, beyond TME resections (extra-visceral), recurrent cancer, and patients with previous local excision were excluded. Subsequently, all staging MRI scans were reviewed by RH, SR and an expert radiologist (AS), in order to only include patients with MRI defined low rectal tumors. In accordance with the LOREC [14] definition of low rectal cancer, a tumor was considered low if the distal border was located distal to the point where the levator ani muscles insert on the pelvic bone on sagittal MRI (Fig. 1).

In an attempt to reduce bias of confounding factors in this observational study, the two groups were matched for nine variables using propensity scores with a tolerance of 0.01, on a one-to-one basis. Independent variables included in the model were sex, age (< 65 or > 65 years), ASA (1–2 or 3–4), BMI (< 30 or > 30 in kg/m^2^), tumor height from anorectal junction (0–1 cm or > 1 cm), clinical TNM-stage (1–2 or 3–4), neo-adjuvant therapy and type of operation (abdominoperineal excision (APE) or anterior resection (AR)). In the unmatched cohort, BMI was not known in five cases and therefore the mean BMI was imputed before matching. The matching was performed using propensity scores derived from a logistic regression model; the dependent variable was operative approach (TaTME or lapTME).

### Outcomes

Primary outcome was resection margin involvement (R1 resection), defined as positive circumferential resection margin (CRM) and/or positive distal resection margin (DRM). Secondary outcomes included further histopathological features (specimen quality, length of circumferential and distal resection margins) and intra- and post-operative outcomes (conversion rate, intra-operative adverse events, 30-day morbidity including anastomotic leakage (AL), 30-day Clavien–Dindo, 30-day readmission rate).

Histopathological assessment of the specimen was carried out by experienced consultant histopathologists. A resection margin was considered involved if the distance of tumor or malignant lymph node to the edge of resection was 1 mm or less. Quality of the TME specimens was categorized using descriptions by Quirke et al. [16].

### Peri-operative course

Preoperative assessment for rectal tumors included colonoscopy with biopsies, MR imaging of the pelvis and CT-scan of the chest, abdomen and pelvis. A multidisciplinary team decided whether patients needed neoadjuvant therapy, usually in the form of long course chemo-radiation. In these cases, a repeat MRI-scan was obtained to assess tumor regression after the neo-adjuvant therapy. All patients were treated according to the UK guidelines for the treatment of rectal cancer [17].

Pre-operatively, patients received mechanical bowel preparation and antibiotic prophylaxis was administered intravenously on the day of surgery. All procedures were performed by the same surgical team consisting of six colorectal surgeons in one hospital. Three of those surgeons started performing TaTME since 2013.

The TaTME technique is thoroughly described in a previously published article by our unit [18]. Transanal conversion was defined as a resection that was not completed transanally as intended, but rather continued further from the abdominal approach; either open, laparoscopic or robotic.

Laparoscopic TME was performed using a multi-port set-up, with completion of TME dissection laparoscopically from above. Conversion was defined as a procedure that was started with the intention to perform a laparoscopic dissection, but was completed as an open resection requiring a midline laparotomy. Decision of fashioning a diverting stoma was made for each patient individually.

Post-operatively, patients followed the enhanced-recovery protocol, introduced in 2015, when appropriate.

### Statistical analysis

All categorical data are presented as number of cases and percentages, whilst continuous data are shown as either mean ± standard deviation (range) or as median and interquartile range (IQR), depending on the data distribution. Data were analyzed using the Statistical Package for Social Sciences (SPSS) of IBM Statistics, version 24.0. Propensity score-matching was carried out as described above. Categorical and continuous variables were compared using a Chi-square test and the Mann–Whitney *U* test, respectively. A Fisher’s exact test was used for variables with less than five observations. A *P* value ≤ 0.05 was considered statistically significant.

## Results

A total of 81 consecutive patients underwent TaTME in the period June 2013 until July 2017, of whom 52 patients met the inclusion criteria. The conventional group consisted of 127 patients with low rectal cancer who underwent laparoscopic TME surgery between August 2006 to December 2016. After propensity score-matching, 41 patients in each group were included for comparison. Patient and tumor characteristics are shown in Table 1, no significant differences were observed between the two groups.

**Table 1:** Patient and tumor characteristics

Factor	Laparoscopic TME 41 cases	TaTME 41 cases	*P* value
Patient characteristics			
Gender, *n* (%)			
Male	32 (78.0)	34 (82.9)	0.577
Female	9 (22.0)	7 (17.1)	
Age in years, mean ± SD (range)	66.0 ± 9.2 (48–83)	62.5 ± 10.7 (33–87)	0.145
ASA score, *n* (%)			
I + II	38 (92.7)	36 (87.8)	0.523
III + IV	3 (7.3)	5 (12.2)	
BMI in kg/m², mean ± SD (range)	26.1 ± 4.0 (19.4–36.0)	26.7 ± 1.9 (20.9–32.3)	0.243
Neoadjuvant therapy, *n* (%)			
No	23 (56.1)	23 (56.1)	0.086
RT	5 (12.2)	0 (0.0)	
Chemo	0 (0.0)	1 (2.4)	
CRT	13 (31.7)	15 (36.6)	
SCRT	0 (0.0)	2 (4.9)	
Tumor characteristics on MRI			
Height from ARJ in cm, median (IQR)	1.5 (0.0–3.0)	2.0 (0.0–4.0)	0.489
Tumor size in mm, median (IQR)	43.0 (37.0–55.0)	46.5 (34.5–53.8)	0.890
Anteriorly located, *n* (%)	19 (46.3)	24 (58.5)	0.447
TNM-staging, *n* (%)			
Stage 1	8 (19.5)	9 (22.0)	1.000
Stage 2	16 (39.0)	15 (36.6)	
Stage 3	13 (31.7)	13 (31.7)	
Stage 4	4 (9.8)	4 (9.8)	
CRM involvement, *n* (%)	19/39 (48.7)	19/41 (46.3)	0.832

CRM involvement on MRI is defined as involved if the distance of tumor or malignant lymph node to the mesorectal fascia was ≤ 1 mm on MRI

Percentages are shown as percentages of the whole group not including missing values

*SD* standard deviation, *IQR* inter quartile range, *ASA* American Society of Anaesthesiologists, *BMI* Body Mass Index, *ARJ* anorectal junction

In both groups, the majority was male, 32 (78.1%) in the laparoscopic group and 34 (82.9%) in the TaTME group (*P* = 0.577). Median tumor height from anorectal junction was 1.5 cm (IQR 0.0–3.0) in the lapTME group and 2.0 cm (IQR 0.0–4.0) in the TaTME group (*P* = 0.489). On pre-operative MRI, the tumor was located anteriorly in 19 patients (46.3%) in the laparoscopic group versus 24 patients (58.5%) in the TaTME group (*P* = 0.447). The CRM was threatened in 19 patients in both groups.

Histopathological outcomes are depicted in Table 2. Resection margin (R1) was positive in 5 cases (12.2%) in the lapTME group versus 2 (4.9%) positive margins, both due to positive CRM, in the TaTME group (*P* = 0.432). In the lap TME group, the resection margin was involved due to a positive CRM in two specimens, a positive DRM in two specimens, and in one specimen, both the CRM and DRM were involved. The quality of the specimen was graded as complete in 84.0% in the laparoscopic group versus 92.7% in the TaTME group (*P* = 0.412). Median tumor distance to the CRM in the lapTME group was 5 mm (IQR 3.0–10.0) versus 10 mm (IQR 4.2–12.0) after TaTME (*P* = 0.065). The mean number of harvested lymph nodes was 14 (IQR 11–24) in the lapTME versus 18 (IQR 13–26) in the TaTME group (*P* = 0.102).

**Table 2:** Histopathological outcomes

Factor	Laparoscopic TME 41 cases	TaTME 41 cases	*P* value
Primary outcome			
R1 resection, *n* (%)	5 (12.2)	2 (4.9)	0.432
Positive CRM	3 ^(1 AR, 2 APE)^	2 ^(2 AR)^	0.675
Positive DRM	3 ^(3 AR)^	0	0.241
Secondary histological outcomes			
Tumor distance to CRM in mm, median (range)	5.0 (3.0–10.0)	10.0 (4.2–12.0)	0.065
Tumor distance to DRM in mm, median (range)	20.0 (9.8–41.3)	20.0 (10.0–40.0)	0.649
Pathological T stage, *n* (%)			
ypT0	2 (4.9)	2 (4.9)	0.809
T1	3 (7.3)	1 (2.4)	
T2	11 (26.8)	13 (31.7)	
T3	25 (61.0)	25 (61.0)	
T4	0 (0.0)	0 (0.0)	
Pathological N stage, *n* (%)			
N0	22 (53.7)	26 (63.4)	0.679
N1	15 (36.6)	11 (26.8)	
N2	4 (9.8)	4 (9.8)	
Specimen quality, *n* (%)			
Complete	21 (84.0)	38 (92.7)	0.412
Minor defects	4 (16.0)	3 (7.3)	
Major defects	0 (0.0)	0 (0.0)	
*Missing*	16	0	
Lymph nodes harvested, median (range)	14 (11–24)	18 (13–26)	0.102

Table 3 presents clinical outcomes. Conversion rate to midline was significantly lower in the TaTME group, namely 0 (0.0%), against 9 (22.0%) conversions in the lapTME group (*P* < 0.001). Median operative time was 300 min (IQR 240–378) in the laparoscopic group and 318 min (IQR 270–375) in the TaTME group (*P* = 0.290). AL rate did not differ significantly and was 14.8% in lapTME and 17.9% in TaTME (*P* = 1.000). All anastomotic leakages occurred within 30 days after index surgery. Hospital stay was 8 days (IQR 7–11) in the TaTME group and 11 days (IQR 8–17) after lapTME (*P* = 0.052). In all TaTME cases, a defunctioning stoma was fashioned. Thirty-day unplanned readmission rate and Clavien–Dindo classification scores did not show any significant differences between the two groups. In the lapTME group, five patients developed a Clavien–Dindo IIIb complication, of which three cases were anastomosis related. In the TaTME group, four of the six patients with a Clavien–Dindo IIIb complication had anastomosis-related problems.

**Table 3:** Clinical outcomes

Factor	Laparoscopic TME 41 cases	TaTME 41 cases	*P* value
Operative time minutes, median (range)	300 (240–378)	318 (270–375)	0.290
Operation type, *n* (%)			
AR	27 (65.9)	28 (68.3)	0.814
APE	14 (34.1)	13 (31.7)	
Intra-operative complications, *n* (%)	3 (7.3)	1 (2.4)	0.616
Conversion (to midline), *n* (%)	9 (22.0)	0 (0.0)	< **0.001**
Defunctioning stoma, *n* (%)	24/27 (88.9)	28/28 (100.0)	0.070
Hospital stay in days, median (range)	11 (8–17)	8 (7–11)	0.052
Unplanned Readmissions within 30 days, *n* (%)	8 (19.5)	6 (14.6)	0.557
Postoperative complications within 30 days, *n* (%)	14 (34.1)	19 (46.3)	0.260
Anastomotic leakage, *n* (%)	4/27 (14.8)	5/28 (17.9)	1.000
Clavien–Dindo 30 days classification, *n* (%)			
None	27 (65.9)	22 (53.7)	0.772
I	3 (7.3)	6 (14.6)	
II	4 (9.8)	4 (9.8)	
IIIa	2 (4.9)	3 (7.3)	
IIIb	5 (12.2)	6 (14.6)	
IV and V	0 (0.0)	0 (0.0)	

Intra-operative complications defined as major complications, including: visceral injury, bleeding, ischaemia. Not including conversion

## Discussion

This propensity score-matching analysis is the first study that compares TaTME with lapTME for low rectal cancer based on a strict anatomical definition. By including only MRI-defined low rectal cancers, we have focused on a highly selected patient group, known to pose greater technical difficulty and increased risk of poorer oncological and clinical outcomes. Moreover, by propensity matching for nine potential confounding factors, homogeneous groups for comparison were created.

This study did not show a significant decrease in R1 resections after TaTME.

However, we were able to demonstrate a significant decrease in conversion rate from 22.0% in the laparoscopic group, to no converted cases in the TaTME cohort (*P* < 0.001). So far, six other comparative matched studies on this topic have been published [19–24], and only the study by Chen and Persiani [20, 24] included more TaTME cases (*n* = 50 and *n* = 46, respectively). However, none of these studies focused specifically on low rectal cancer. Similarly to our findings, Persiani et al. also reported a significant decrease in conversion rates in TaTME versus lapTME, from 19.6 to 0%, respectively. Low conversion rates for TaTME were also reported in the recent update from the International TaTME registry (4.3%) and the largest systematic review to date by Deijen et al. (3%) [25, 26]. The study by Oostendorp et al. [27] confirmed the promising conversion rates in TaTME, as the authors report on a conversion rate of 2% compared to 13.7% in a comparative cohort of four recent large RCT’s on lapTME [7–9, 28]. The conversion rate in our comparative laparoscopic cohort was 22%, which is comparable to other studies describing high conversion rates in laparoscopic TME, ranging from 12 to 50% [29–31]. The low conversion rates observed in TaTME have the potential to improve oncological outcomes, as several studies found increased risk of loco-regional recurrences and decreased disease-free survival rates in patients after converted laparoscopic procedures [11, 12]. Moreover, decreasing the conversion rate in TaTME also influences the long-term morbidity with a lower rate of incisional hernias and adhesional obstructions [32].

The hypothesis that TaTME improves positive margin rates was not confirmed in this study, although there appeared to be a trend towards lower R1 rates (from 12.2 to 4.9%). The studies by Chen, De’Angelis, Marks and Velthuis et al. [21–24] showed a similar trend in decreased rate of positive margins after TaTME, although also not significant. The only comparative study reporting on a significant decrease in involved margins was the study by Chang and coworkers [19]; 0 of 23 cases in TaTME versus 4 of 23 cases after lapTME (*P* = *0.037*). The reported positive resection margin rates in the first two reports from the international TaTME registry based on 634 and 1540 TME resections for cancer, were 2.7% and 4.9%, respectively [25, 33]. The systematic review by Deijen et al. [26] analyzing 794 cases from 33 studies, reported a positive margin rate of 4.7%. These results suggest that TaTME has the potential to decrease R1 rate, an important predictor of LR [34], when compared to lapTME, as three recent randomized controlled trials found R1 rates in laparoscopic TME surgery varying from 7 to 12% [7–9].

In this study, we matched for nine factors that are known to increase case difficulty and subsequently influence the risk of R1 resection. The Mercury II study group recently published a stratification model of pre-operative features on MRI that influence development of local recurrence after resection for rectal cancer [35]. Those risk factors on MRI are extra-mural vascular invasion (EMVI) status, an anteriorly located tumor, predicted CRM involvement and tumor height < 4 cm from anal verge. Correspondingly, we matched for tumor height < 1 cm from the anorectal junction. We were not able to match for the other factors; however, our baseline characteristics show that these variables were comparable between both groups, except for EMVI status, which was not routinely reported for all patients early on in the control group.

Over the past decades, surgical management for rectal cancer has changed. Nevertheless, whether by open or laparoscopic surgery, TME for low tumors encounters many challenges, which can be potentially overcome with TaTME. When approaching from below, patient characteristics seem to have less impact on the procedure, as a multivariate analysis in the study by Penna et al. [33], failed to show any patient characteristics to be of influence on the risk of poor specimen outcome in TaTME. Also, a clearer visualization may result in less traction on the specimen and more accurate TME dissection, possibly providing better quality TME and negative margins, a more accurate distal transection, and lower conversion rates compared to laparoscopic surgery. Furthermore, the technique allows for a double pursestring anastomosis, avoiding difficulties with stapling the rectal stump, and therefore may have the added potential to lower the anastomotic leak rate [36, 37].

This hypothesis on AL was not seen in our results. The AL rate of 18% in our TaTME cohort might seem relatively high. However, when interpreting these rates, tumor height must be taken into account. A large study by Borstlap et al. [38] found an early AL rate of 13.4% in patients who underwent low anterior resection, but this increased to 20% beyond 30 days. An independent risk factor for AL was a distal tumor (≤ 3 cm from ARJ), with an odds ratio of 1.88. The study by Bertelsen and coworkers [39] published on a five to six time increase in AL risk for low rectal tumors. The recent registry study on anastomotic leaks in TaTME by Penna et al. [25] published on an early AL rate of 7.8%, which is within an acceptable range compared to previously reported incidences in colorectal surgery [40].

The learning curve effect regarding the new TaTME approach could be the reason why more significant differences were not seen favoring TaTME, in particular for histopathological outcomes and anastomotic leakage, but will hopefully improve with increasing surgeon experience. TaTME is not an easy surgical technique, and surgeons have to adapt to the unfamiliar anatomical view from below. Our institution regularly receives visitors and is a training center for surgeons aiming to learn TaTME. This might be the reason that operative time was not decreased in the TaTME group, while other comparative studies [22, 41–43] have reported on the ability of TaTME to effectively decrease operative time when performed with a two-team approach.

We acknowledge that this study has limitations. First, the retrospective view on prospectively collected data. By propensity score-matching for nine factors known to make a procedure more difficult, we attempted to create two comparable groups; nevertheless, we could not match for unknown confounders. Further, a large confounder in this study is time, as the inclusion period for the conventional cohort starts in 2006. This may have negatively influenced the outcomes of this group, as surgeons were still on their learning curve for laparoscopic TME, particularly in the early part of this period. However, we compared results to TaTME from the start of implementing this technique in our institution, and so included the early stages of the surgeons learning curve for both procedures. We could not account for possible other improvements in management over time, which might have skewed results. Third, the sample size is relatively small, resulting in a lack of statistical power. Finally, this study only reports on short-term outcomes. Currently, the literature on TaTME still lacks long-term oncological and functional data. Longer follow-up, on large patient cohorts, is needed to provide such data, hence the importance of the international TaTME registry [14]. Recently, an international randomized clinical trial (COLOR III [44]) comparing transanal to laparoscopic TME for rectal cancers started patient recruitment and will be completed in the next 5 to 8 years. While awaiting these results, non-randomized comparative studies are the best level of evidence available.

This study aimed to compare results of TaTME with lapTME in a highly selected patient group by only including MRI-defined low rectal tumors. No significant difference was found in R1 rate. This novel technique warrants further experience and prospective randomized studies to ensure oncologic safety and assess long-term functional results.

## Appendix

Abbreviations and definitions.

**Table:**

TaTME	Transanal total mesorectal excision
APE	Abdominoperineal extirpation
AR	Anterior resection
R1	Microscopic presence of tumor cells at the distal or circumferential resection margins or within a lymph node < 1 mm from the mesorectal fascia of the excised specimen
CRM [+]	Circumferential resection margin of the excised specimen [presence of tumor cells within 1 mm from the excised non-peritonealised surface of the rectum]
DRM [+]	Distal resection margin of the excised specimen [presence of tumor cells within 1 mm from the excised distal end of the specimen]
Quality of TME specimen	Using the Quirke grading system for completeness of mesorectal dissection, each TME specimen is graded as having either an intact mesorectum, minor or major defects.^17^
TNM-stage	Classification of colorectal carcinoma (tumor, lymph nodes, metastasis)
ARJ	Anorectal junction (located approximately 3 cm proximal from AV)
AV	Anal valve
EMVI	Extra-mural vascular invasion (sign seen on MRI-imaging)
Clavien–Dindo classification	Ranking classification of postoperative complications, based on the therapy used for that specific complication
1	Any deviation from the normal postoperative course without the need for pharmacological treatment or surgical, endoscopic and radiological interventions
2	Requiring pharmacological treatment with drugs other than such allowed for grade I complications
3a	Requiring surgical, endoscopic or radiological intervention not under general anesthesia
3b	Requiring surgical, endoscopic or radiological intervention under general anesthesia
4	Life-threatening complication requiring IC/ICU-management
5	Death of a patient

*Source* http://www.assessurgery.com/clavien-dindo-classification/
